# Etoposide Hypersensitivity Reactions in Pediatric Oncology: Understanding Mechanisms and Optimizing Management

**DOI:** 10.3390/cancers18071141

**Published:** 2026-04-01

**Authors:** Maria De Filippo, Anna Maria Zicari, Bianca Laura Cinicola, Martina Capponi, Luciana Granata, Francesca Stefanachi, Silvia Carosini, Francesca Ferrozzi, Loredana Amoroso

**Affiliations:** 1 Pediatric Onco-Hematology Unit, Department of Maternal Infantile and Urological Sciences, Policlinico Umberto I, Sapienza University of Rome, 00161 Rome, Italy; m.capponi@policlinicoumberto1.it (M.C.); l.granata@policlinicoumberto1.it (L.G.); f.stefanachi@policlinicoumberto1.it (F.S.); l.amoroso@policlinicoumberto1.it (L.A.); 2 Allergy and Immunology Pediatric Unit, Department of Maternal Infantile and Urological Sciences, Policlinico Umberto I, Sapienza University of Rome, 00161 Rome, Italy; a.zicari@policlinicoumberto1.it (A.M.Z.); bianca.cinicola@uniroma1.it (B.L.C.); 3 Department of Radiological, Oncological and Pathological Sciences, Sapienza University of Rome, 00161 Rome, Italy; 4 Pharmacy Unit, Policlinico Umberto I, Sapienza University of Rome, 00161 Rome, Italy; s.carosini@policlinicoumberto1.it (S.C.); f.ferrozzi@policlinicoumberto1.it (F.F.)

**Keywords:** pediatric cancer, precision medicine, allergic hypersensitivity reaction, etoposide hypersensitivity reactions, polysorbate-80, ethanol toxicity, desensitization protocol

## Abstract

Etoposide is a key component of many frontline and salvage chemotherapy regimens in pediatric oncology. However, its clinical use may be complicated by acute hypersensitivity reactions, which can disrupt treatment continuity and compromise therapeutic efficacy. Recent pediatric studies indicate that the incidence of etoposide-associated hypersensitivity is higher than historically reported and varies according to disease type and administration conditions. This narrative review provides a comprehensive overview of etoposide hypersensitivity in children, integrating clinical, immunological, and pharmaceutical perspectives. Current evidence suggests that true IgE-mediated type I hypersensitivity is uncommon in pediatric patients, whereas most reactions are consistent with non-IgE-mediated mechanisms, often related to formulation excipients such as polysorbate 80 and ethanol. We discuss the clinical features and differential diagnosis of acute reactions associated with etoposide administration, including hypersensitivity reactions and infusion-related reactions, such as ethanol-related toxicity. In addition, modifiable risk factors, preventive measures, and management strategies—such as infusion optimization, alternative formulations, and rapid desensitization protocols—are critically reviewed. A mechanism-based approach to etoposide hypersensitivity is essential to ensure patient safety while maintaining treatment intensity in pediatric cancer care.

## Simple Summary

Etoposide is widely used to treat childhood cancers, but some children experience acute reactions during intravenous infusion. Recent evidence shows that these reactions occur more often than previously thought in pediatric age. Most reactions start shortly after infusion begins and involve skin or breathing symptoms. Importantly, confirmed IgE-mediated allergic reactions appear to be rare. In most cases, acute reactions are more consistent with non-IgE-mediated mechanisms or infusion-related reactions, potentially associated with formulation components such as polysorbate 80 or the pharmacologic effects of ethanol. Correctly identifying the type of reaction is crucial because management differs. Adjusting infusion conditions or switching to alternative formulations can often prevent recurrence, while desensitization protocols allow safe continuation of treatment when necessary. Understanding the mechanisms underlying these reactions helps avoid unnecessary discontinuation of an essential anticancer drug and supports safer, more effective therapy for children with cancer.

## 1. Introduction

Etoposide (VP16) is a semi-synthetic podophyllotoxin analogue and represents a cornerstone of several contemporary chemotherapeutic regimens used in pediatric oncology for the treatment of both solid tumors and hematologic malignancies. Together with teniposide, etoposide belongs to the class of topoisomerase II inhibitors, which exert their cytotoxic effect by stabilizing the enzyme–DNA cleavage complex and preventing DNA strand religation, leading to irreversible DNA damage and apoptosis in rapidly proliferating malignant cells. Although the two agents share a common mechanism of action, structural differences—most notably the substitution of a thenylidene group in teniposide—may account for differences in pharmacokinetics and toxicity profiles. Importantly, clinically relevant cross-reactivity between etoposide and teniposide has not been consistently demonstrated. Clinically, VP16 is widely used—either alone or combined with other cytotoxic agents—in treating a broad range of pediatric malignancies, including leukemias, lymphomas, sarcomas, neuroblastoma, rhabdoid tumors, and germ cell tumors. It is available as both an intravenous (IV) and an oral formulation, which increases its versatility across treatment settings. Since its approval by the U.S. Food and Drug Administration in 1984, etoposide has remained a fundamental component of pediatric cancer therapy, contributing significantly to improved survival outcomes. Nevertheless, its use is limited by a range of adverse effects, among which hypersensitivity reactions are of particular concern due to their unpredictable onset and potential to interrupt or delay chemotherapy delivery [1,2,3]. Although early studies indicated a low overall rate of etoposide hypersensitivity reactions (HSRs), around 0.7–3%, more recent pediatric studies report higher rates ranging from 5% to 15% [4,5], with substantial variability across studies. Notably, the incidence of HSRs differs according to the underlying malignancy, with the highest rates observed in children with Hodgkin lymphoma and acute lymphoblastic leukemia (up to 51% and 46%, respectively), and intermediate rates of approximately 10–20% in solid tumors [6,7,8]. This variability likely reflects not only differences in patient populations but also heterogeneity in the definition of hypersensitivity, which in some studies refers mainly to immediate IgE-mediated (type I) reactions, whereas in others includes a broader spectrum of acute reactions associated with drug administration, including infusion-related and non-IgE-mediated reactions (Table 1).

In accordance with the EAACI nomenclature of allergic diseases and hypersensitivity reactions, the term hypersensitivity reaction (HSR) is used as a clinical umbrella term referring to reproducible symptoms or signs triggered by exposure to a stimulus tolerated by normal individuals. Within this framework, hypersensitivity reactions may include IgE-mediated allergic reactions (type I hypersensitivity) as well as non-IgE inflammatory responses to chemical substances, discussed within the EAACI framework as type VII hypersensitivity [9]. In contrast, infusion-related reactions, such as those related to the pharmacologic effects of ethanol, represent a broader clinical category used in oncology to describe reactions occurring during drug administration that are not necessarily mediated by hypersensitivity mechanisms.

From a pharmaceutical perspective, the IV formulation of etoposide presents significant pharmaceutical challenges primarily related to its limited aqueous solubility and propensity for physical instability. Because of this unfavorable physicochemical profile, intravenous etoposide is formulated using a cosolvent system containing polysorbate 80 (PS80), polyethylene glycol (PEG 300), and ethanol, which enables parenteral administration by maintaining the drug in solution. Although an oral formulation of etoposide is available, intravenous administration is frequently preferred in many oncology protocols because it provides more predictable systemic exposure. In contrast, oral etoposide exhibits variable bioavailability (approximately 25–75%) and considerable interindividual pharmacokinetic variability, which may result in less predictable drug exposure [10,11]. However, despite the presence of these excipients, IV etoposide remains susceptible to precipitation following dilution, particularly when higher concentrations are used, when sodium chloride solutions are employed as diluents, or when the solution is exposed to low temperatures or prolonged storage times. To reduce the risk of precipitation, regulatory agencies and manufacturers recommend diluting etoposide to a final concentration generally not exceeding 0.2 mg/mL. Although higher concentrations, up to 0.4 mg/mL, have occasionally been reported in clinical practice, these conditions are associated with an increased likelihood of crystal formation and therefore require strict control during preparation, storage, and administration. Given the well documented tendency of etoposide to precipitate during intravenous infusion, the use of in-line filtration is recommended during IV administration [12]. In-line filters (0.22–0.45 μm) are used to retain precipitated particles and reduce infusion-related risks, as supported by regulatory documents and compatibility studies [13]. In addition to pharmaceutical and technical considerations, appropriate management of etoposide administration is of clinical relevance in pediatric oncology. The exclusion of etoposide from chemotherapy regimens, or the need to substitute it with less effective treatments due to hypersensitivity reactions, may prevent pediatric patients from receiving an essential antineoplastic agent. Moreover, alternative drugs or therapeutic regimens may be associated with reduced efficacy, increased toxicity, or higher costs, potentially compromising both clinical outcomes and healthcare resource utilization [14,15].

**Figure 1:**
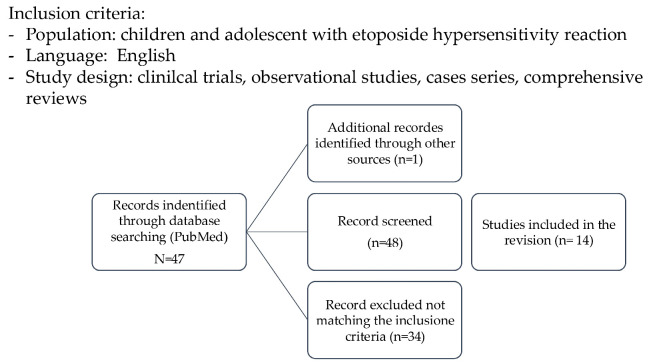
Flowchart of Literature Search and Study Selection.

The aim of this narrative review is to provide a comprehensive and critical synthesis of current evidence on etoposide hypersensitivity in pediatric patients. Specifically, we seek to (i) define the incidence and clinical spectrum of acute reactions associated with etoposide administration, including hypersensitivity reactions and infusion-related reactions; (ii) clarify the immunologic and non-immunologic mechanisms involved; (iii) identify modifiable risk factors related to formulation and administration; and (iv) evaluate preventive strategies and therapeutic approaches, including desensitization protocols, that allow safe continuation of etoposide therapy. By integrating mechanistic insights with practical clinical recommendations, this review aims to support personalized management strategies and optimize the safe use of etoposide in pediatric oncology. A review of articles was conducted using the online database PubMed, combining the terms “etoposide” AND “hypersensitivity reaction” OR “allergic reaction” AND “children”. The literature review was performed in September 2025 and included publications from 1990 to 2025. All studies meeting the following criteria were included: (i) case reports, clinical trials, cross-sectional, and cohort studies published in English in peer-reviewed journals, and (ii) where participants were children with hypersensitivity reactions to etoposide. Potentially eligible publications were manually screened and reviewed, and no relevant publications were excluded (Figure 1).

**Table 1:** Summary of Pediatric Studies Reporting Incidence, Presentation, and Outcomes of Etoposide Hypersensitivity.

Author and Year	Country	Study Years	Sample Size	Underlying Diagnosis of Children with Etoposide HSR	Etoposide HSR Pediatric Patients	Cycle/Dose of HSR Onset	Cumulative Incidence of Etoposide HSR	Life Threatening Reactions *	Outcome
McBride P et al., 2024 [8]	USA	2021–2023	Children: 150	73.3% patients with classical Hodgkin lymphoma	Total 15 Mean age: 195 months (≈16.3 years) (range 6.1–20.7 years)	N.A.	10%	N.A.	2 patients tolerated subsequent doses with slower infusion + premedication 12 switched to etoposide phosphate with 0 recurrences 1 discontinued all etoposide formulations
Kelly Dodier et al., 2023 [16]	Canada	2015–2018	Total: 284 Children: 40	Heterogeneous pediatric cancers (hematologic malignancies and solid tumors); no single diagnosis predominated	Total: 8 6/23 (26.1%) during ILF use vs. 2/17 (11.8%) without ILF Mean age 8.6 ± 6.4	Cycle 1: similar with and without ILF (5 reactions total across all ages) Cycles 2–3: reactions only occurred during ILF use	12.2% with ILF vs. 5% without ILF (*p* = 0.09)	0	Successful etoposide rechallenge Change to etoposide phosphate or to oral etoposide Change to etoposide-free protocol
Báez Gutiérrez N et al., 2022 [5]	Spain	2013–2020	Children: 213	Heterogeneous pediatric cancers (hematologic malignancies and solid tumors); no single diagnosis predominated	Total: 23 Mean age: N.A.	N.A.	10.8%	0	Successful etoposide rechallenge with premedication and adjustments to infusion rate
Emma M. Tillman et al., 2021 [17]	USA	2010–2020	Children: 3445	Predominantly Hodgkin lymphoma and neuroblastoma, plus other pediatric cancers	Total: 32 Mean age 8.5 ± 5.8 years	53% occurred at first dose (17/32)	Overall: 1%; Without ILF: ~1%; With ILF: 13% at CMH (28 reactions/566 pts)	7 (22%)	41% rechallenged → all tolerated at least one future dose 69% switched to etoposide phosphate with 0 reactions Reactions prevented by slower infusion and/or premedication
Rebecca Ronsley et al., 2021 [18]	Canada	2013–2018	Children: 192	Heterogeneous pediatric cancers (hematologic malignancies and solid tumors); no single diagnosis predominated	Total: 39 Mean age: N.A.	N.A. as cycle number Pre-filter: 2 reactions; During filter use: 33 reactions; Post-filter: 1 reaction.	Type I HSR: 5%; Anaphylaxis: 3%	11 (28%)	Three patients rechallenged had repeat HSRs;
Irem Turgay Yagmur et al., 2020 [19]	Turkey	2007–2019	Children: 519	Heterogeneous pediatric cancers (hematologic malignancies and solid tumors); no single diagnosis predominated	Total: 7 Mean age: N.A.	2 at the first dose 1 at the second 2 at the third 1 at the fourth 1 at the sixth	1.35%	4 (57%)	Successful etoposide rechallenge with premedication and adjustments to infusion rate: 2 patients Desensitization protocol: 3 patients Intravenous to oral etoposide switch: 1 patient Change to etoposide-free protocol:1 patient
Winifred M Stockton et al., 2020 [20]	USA	2012–2017	Children: 246	Heterogeneous pediatric cancers (hematologic malignancies and solid tumors); no single diagnosis predominated	Total: 52 Mean age 10.8 ± 6.6 years	Reactions occurred earlier without titration (~2.8 doses) vs with titration (~5 doses)	27.1%	3 (6%)	Switch to etoposide phosphate successful in 97.4% (1 recurrence) Desensitization successful in 88.9% (1 recurrence)
Melissa M. Hudson et al., 1993 [7]	USA	1990–1992	Children: 45	Hodgkin’s lymphoma	Total: 23 Median age 15 years (range, 8 to 18)	First dose (13 pts), second dose (5), third dose (2), seventh (1), 11th (1), 12th (1). Most reactions after dose 1–2; median time to reaction 5 min	51%	3 (13%)	78% tolerated re-challenge with slower infusion +/− premedication 5 had recurrent reactions 3 cross-reacted with teniposide
Kellie SJ et al., 1991 [6]	USA	1984–1985	Children: 88	Acute lymphoblastic leukemia (ALL)	Total: 78 reactions	- First-dose reactions: 3/88 patients (3.4%) - Peak risk: cumulative dose 2000–3000 mg/m^2^	34%	0	Successful etoposide rechallenge with premedication and adjustments to infusion rate

* According to Common Terminology Criteria for Adverse Events, a life-threatening reaction must be considered ≥4 grade. HSR: hypersensitivity reactions. ILF: in-line filter. N.A.: not available.

## 2. Pathophysiological Overview of Etoposide Hypersensitivity in Pediatric Patients

In pediatric oncology, hypersensitivity reactions (HSRs) to etoposide comprise a heterogeneous spectrum of immediate-onset adverse events, the underlying mechanisms of which remain incompletely understood. In general, both immunologic and non-immunologic pathways are known to contribute to the development of HSRs to chemotherapeutic agents, and a deeper understanding of these mechanisms is crucial to guide preventive strategies and enable the safe re-administration of essential antineoplastic drugs. According to the updated EAACI classification (European Academy of Allergy and Clinical Immunology) of hypersensitivity disorders, these reactions can be broadly divided into type I reactions, corresponding to IgE-mediated allergic hypersensitivity, and type VII reactions, defined as direct cellular and inflammatory responses to chemical substances [9]. Although both mechanisms have been described, several data indicate that type VII reactions appear to predominate in children receiving intravenous etoposide, whereas true IgE-mediated type I allergy appears to be relatively uncommon [19,20,21,22,23]. Basophils and mast cells are the principal effector cells involved in immediate-type HSRs and may be activated through either IgE-mediated or non-IgE-mediated mechanisms [22,23]. In IgE-mediated reactions, drug-specific IgE antibodies bound to the high-affinity IgE receptor (FcεRI) on the surface of basophils and mast cells undergo cross-linking upon re-exposure to the culprit drug, triggering cellular activation and the release of mediators such as histamine and tryptase, ultimately leading to clinical manifestations of anaphylaxis [22,23]. IgE-mediated basophil activation is associated with the upregulation of specific surface markers, including CD203c and CD63, which can be quantified by flow cytometry and constitute the biological basis of the basophil activation test (BAT) [23]. CD203c is expressed at low levels on resting basophils and is rapidly upregulated following activation, whereas CD63 becomes detectable on the cell surface after fusion of intracellular granules with the plasma membrane during degranulation [23]. However, basophils can be activated not only through IgE/FcεRI cross-linking but also via IgE-independent pathways, including stimulation by complement anaphylatoxins (e.g., C3a, C5a), IgG immune complexes via Fcγ receptors, and other receptor-mediated or pharmacologic stimuli [23]. Accordingly, activation markers such as CD63 and CD203c reflect basophil activation but are not specific for IgE-mediated mechanisms [23]. Therefore, the basophil activation test (BAT) should be considered a functional assay of cellular reactivity, and its results must be interpreted in conjunction with clinical history, skin testing, and the suspected underlying mechanism [22,23]. Recent BAT studies in adult patients report basophil activation in up to 80% of cases with a clinical history of immediate reactions to etoposide; however, this likely reflects overall basophil reactivity rather than confirmed IgE-mediated sensitization and may include IgE-independent mechanisms [24]. Notably, discordant results between in vivo skin testing and in vitro basophil activation have been described, highlighting the inherent limitations and lack of standardization of current diagnostic approaches for antineoplastic drug allergy. While positive skin test results to etoposide have been described in isolated cases, negative findings do not reliably exclude IgE-mediated sensitization. In this context, BAT may represent a useful complementary diagnostic tool, particularly when skin testing is inconclusive, contraindicated, or technically challenging. Although further validation and methodological standardization—especially in pediatric populations—are required, available data suggest that BAT has the potential to improve the identification of patients with true etoposide hypersensitivity reactions and to contribute to a more accurate characterization of the underlying immunologic mechanisms [23,24]. Evidence supporting a confirmed IgE-mediated mechanism in pediatric etoposide hypersensitivity is limited. At present, no validated criteria specifically exist to definitively classify reactions to etoposide administration as IgE-mediated sensitization [22,23]. Pediatric cases suggestive of this mechanism include those with positive skin prick or intradermal tests to etoposide, reproducibility of symptoms upon re-exposure, and successful management through rapid desensitization protocols, which generally indicate an IgE-dependent pathway. To date, only Babaie et al. have provided documented evidence of IgE-mediated hypersensitivity reactions to etoposide confirmed by positive skin testing, as demonstrated in two children who developed immediate anaphylactic reactions during infusion [21].

In the first case, a 10-year-old girl undergoing therapy for anaplastic T-cell lymphoma experienced grade 3 anaphylaxis, presenting with urticaria, angioedema of the lips and tongue, cough, and dyspnea shortly after starting the etoposide infusion. Four weeks later, her allergologic evaluation revealed a positive intradermal test (9 mm wheal at 1:100 dilution of 1 mg/mL of etoposide), despite a negative skin prick test, providing strong evidence of IgE-mediated sensitization to etoposide. She then underwent the standardized rapid drug desensitization protocol; although she developed mild breakthrough symptoms at step 12, the infusion was temporarily slowed, allowing her to complete the procedure successfully during step 11. Importantly, she continued to tolerate the same 11-step desensitization protocol during all subsequent etoposide administrations without significant adverse reactions, demonstrating the reproducibility and safety of repeated desensitization in confirmed IgE-mediated etoposide hypersensitivity. Similarly, the second case involved an 8-year-old girl with relapsed Wilms tumor, who developed grade 2 anaphylaxis manifested by flushing, angioedema, and hypotension during etoposide infusion. Her subsequent evaluation demonstrated a positive intradermal test (8 mm wheal at 1:1000 dilution of 1 mg/mL of etoposide), again consistent with an IgE-mediated mechanism. She tolerated etoposide after a 12-step desensitization procedure on day 1 of each treatment cycle and maintained tolerance during subsequent administrations. In contrast, all other pediatric reports—although documenting immediate reactions and successful desensitization—lack positive skin tests or in vitro markers of IgE sensitization, making an IgE-mediated mechanism only hypothetical. Most reactions are more consistent with non-IgE-mediated immediate reactions, potentially explained by mechanisms described within the EAACI framework of type VII hypersensitivity, rather than classical type I hypersensitivity. In this context, compelling evidence indicates that PS80, the major solubilizer present in conventional IV etoposide formulations, plays a central pathogenic role. Polysorbate 80 is known to cause direct mast-cell degranulation, complement activation, and dose-dependent histamine release, producing a clinical picture that mimics anaphylaxis but lacks the immunologic hallmarks of IgE mediation [25,26]. Several pediatric studies support this mechanism, highlighting the absence of HSRs in children treated with etoposide phosphate or oral etoposide—formulations that do not contain polysorbate 80—as well as the efficacy of preventive strategies such as slower infusion rates and premedication. Notably, these interventions are unlikely to alter the course of a true IgE-mediated reaction, yet they significantly attenuate non-immunologic mast-cell activation, further reinforcing the role of PS80 in the pathogenesis of most immediate reactions to intravenous etoposide [5,16,20,27].

## 3. Clinical Features of Hypersensitivity and Infusion-Related Reactions to Etoposide

In pediatric oncology, HSRs to etoposide usually develop within minutes of the first infusion in up to 50% of cases [7,8,9,10,11,12,13,14,15,16,17,19,20,21,22,23,24,25,26,27]. These reactions typically feature a cluster of mast cell-mediated symptoms, such as flushing, itching, hives, angioedema, cough, difficulty breathing, wheezing or bronchospasm, tachycardia, hypotension, and sometimes gastrointestinal discomfort. The most frequent manifestations of etoposide HSRs are cutaneous symptoms—particularly flushing and urticaria—followed by respiratory features such as dyspnea and cough [28]. Based on current knowledge, no delayed hypersensitivity reactions, including severe cutaneous adverse reactions such as Stevens–Johnson syndrome or toxic epidermal necrolysis, have been reported in association with etoposide. Overall, symptoms can vary from mild to life-threatening and are generally classified within the Common Terminology Criteria for Adverse Events (CTCAE) v6.0 as Allergic Reaction/Hypersensitivity and Anaphylaxis, which reflect either immune or non-immune hypersensitivity responses (Table 2) [29]. Although most reactions are mild to moderate, several pediatric studies have documented severe immediate reactions to etoposide, including anaphylaxis. In a group of 519 children, Turgay et al. identified 4 cases of anaphylaxis, while Tillman et al. reported 7 cases among 32 patients, indicating that serious reactions are rare but possible. Importantly, in all published pediatric cases, symptoms resolved after prompt stopping of the infusion and appropriate drug treatment, with no reported deaths due to etoposide hypersensitivity [17,19]. Importantly, not all reactions attributed to etoposide represent true hypersensitivity and CTCAE also includes other adverse effects that are mechanistically different and should not be mistaken for hypersensitivity, such as myelosuppression, hair loss, vomiting, diarrhea, constipation, mucositis, and loss of appetite. While hypersensitivity reactions are recognized as adverse effects of etoposide, recent pediatric evidence has highlighted that a substantial proportion of infusion-related adverse events attributed to etoposide may in fact reflect ethanol-related infusion reactions rather than true hypersensitivity, introducing clinically relevant diagnostic ambiguity. Conventional intravenous formulations of etoposide contain substantial amounts of ethanol as a solubilizing excipient, leading to clinically relevant alcohol exposure during treatment. This represents a particular concern in pediatric patients, whose enzymatic capacity for ethanol metabolism is immature and highly variable, especially in infants and very young children [30]. Ethanol-related infusion reactions may manifest with neurological and cardiovascular symptoms such as drowsiness, dizziness, altered consciousness, tachycardia, hypotension, hypoglycemia, and metabolic acidosis, which may overlap clinically with infusion-related reactions but arise from a distinct, non-immunological mechanism. The relevance of this issue has been underscored by Claraz et al., who reported that many adverse events during high-dose etoposide infusions in children undergoing hematopoietic stem cell transplant (HSCT) were actually due to ethanol intoxication rather than hypersensitivity. Among 48 children, 41% experienced infusion-related symptoms; however, only 4.1% had hypersensitivity reactions, whereas 37.5% showed signs consistent with ethanol toxicity [31]. Ethanol intoxication typically presented with drowsiness, dizziness, rapid heartbeat, and low blood pressure, without the skin or respiratory signs typical of hypersensitivity, and was more common in children aged 7.5 years or older. Symptoms resolved rapidly, with a median duration of approximately 4 h, and were managed with supportive care, including intravenous hydration, antiemetic therapy, and analgesics. In contrast, true hypersensitivity reactions were characterized by tachycardia, hypotension, cough, oxygen desaturation, cutaneous erythema, and vomiting, required supportive management including intensive care unit (ICU) monitoring, and resolved within 24 h. The higher frequency of reported symptoms in children older than 7.5 years is likely influenced, at least in part, by the retrospective design of the study, as ethanol-related manifestations are often nonspecific and may be under-recognized or under-reported in younger patients. The potential risk of ethanol-related infusion reactions is particularly relevant in patients with low body weight, especially those weighing less than 10 kg, in whom the relative ethanol dose per kilogram may be disproportionately high and clinically significant even at standard dosing. According to European regulatory guidance on ethanol as an excipient in medicinal products, clinically relevant thresholds have been defined to improve risk awareness and patient safety, with 3 g of ethanol per administration commonly regarded as a critical cut-off above which specific warnings and precautionary measures are required [32]. However, this threshold represents a regulatory safety framework rather than a defined toxic dose, and clinically significant effects may occur at lower exposures, particularly in vulnerable pediatric populations such as infants and children with low body weight. This framework is particularly pertinent in pediatric oncology, as several intravenously administered anticancer drugs—including etoposide—contain ethanol in quantities that may substantially exceed this threshold, especially when high doses or prolonged infusions are used. Conventional intravenous formulations of etoposide contain approximately 30% ethanol as a solubilizing excipient [33]. Depending on the administered dose and the specific commercial preparation, intravenous etoposide may correspond to an estimated ethanol exposure in the order of 0.03–0.05 g/kg per infusion in pediatric patients, with relatively higher relative exposure in infants and lowweight children. In children, ethanol exposure is of specific concern because intravenous administration bypasses gastrointestinal first-pass metabolism and because ethanol elimination relies on enzymatic pathways that show marked developmental and inter-individual variability [34]. In early life, the activity of alcohol and aldehyde-metabolizing enzymes is immature, and variability in aldehyde dehydrogenase (ALDH) function—potentially influenced by genetic polymorphisms—may further modulate susceptibility to acetaldehyde accumulation and toxicity. Updated treatment strategies and supportive care recommendations advocate minimizing excipient-related toxicity whenever feasible, particularly in vulnerable populations such as infants and children with low body weight [35]. In patients weighing less than 10 kg, this principle is reflected in international pediatric oncology protocols, including infant acute lymphoblastic leukemia regimens, which stress careful consideration of formulation choice and allow or favor the use of alcohol-free alternatives when available [36]. In this context, etoposide phosphate, a water-soluble prodrug that does not require ethanol, polyethylene glycol, or polysorbate 80 for solubilization, represents a rational option to reduce solvent exposure while maintaining therapeutic efficacy [18,37].

## 4. Risk Factors and Management

**Figure 2:**
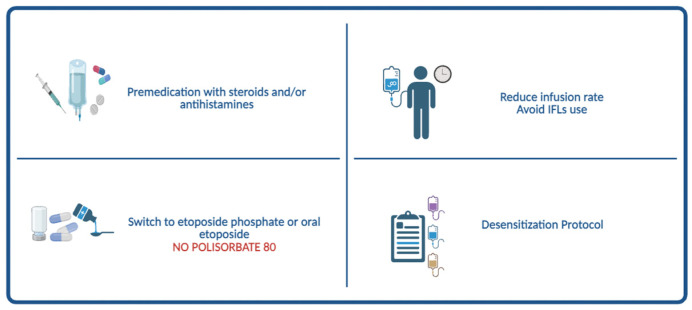
Preventive strategies for etoposide hypersensitivity.

Several pediatric studies have described the risk of HSRs during etoposide administration, highlighting specific risk factors and providing evidence-based management strategies. Across these studies, infusion rate has consistently emerged as a modifiable risk factor for etoposide hypersensitivity. In the cohort described by Stockton et al., children who experienced hypersensitivity reactions received significantly higher infusion rates than those without reactions, prompting the institution to adopt slower infusion protocols [20]. Similarly, other pediatric centers have reported a reduction in recurrent reactions after extending infusion durations to 90–120 min [17]. These findings support the recommendation that etoposide should be infused over at least 60 min, with longer durations preferred for patients with previous reactions. Another significant factor highlighted by recent evidence is the use of in-line filters (ILFs) during etoposide infusion. In a pediatric cohort of 192 children receiving 486 intravenous infusions, Ronsley et al. found that the introduction of 0.22 µm hydrophilic ILFs was linked to a notable increase in hypersensitivity reactions: although the reaction rate was only 1% in both the pre- and post-filter periods, 92% of all hypersensitivity events occurred during filter use, showing a statistically significant rise in risk (pre-filter: Z = 3.978, *p* < 0.001; post-filter: Z = 3.335, *p* < 0.001) [18]. In a larger combined adult and pediatric group of 284 patients, Dodier et al. reported an overall cumulative incidence of etoposide-related hypersensitivity reactions of 9.9%; the incidence tended to be higher during ILF use compared to the combined pre- and post-ILF periods (12.2% vs. 5.2%), and among patients receiving multiple cycles, ILF use increased the risk of hypersensitivity reactions almost fourfold (15.0% vs. 3.9%, *p* = 0.01) [16]. Based on these converging data, the Italian Medicine Agency (AIFA), in coordination with the European Medicine Agency (EMA) and several national agencies, has updated product information to indicate that the use of ILFs during etoposide infusion is linked to an increased risk of hypersensitivity reactions and should therefore be avoided [37]. Several mechanistic hypotheses may explain this association. Filtration can destabilize polysorbate-80 micelles or alter the excipient profile of the formulation, increasing the proportion of free surfactants capable of triggering non-IgE-mediated mast-cell activation. At the same time, mechanical stress across the filter membrane may cause micelle disruption or particulate formation, processes that could enhance complement activation and further contribute to acute infusion-related reactions [12,13]. No studies have specifically evaluated the interaction between in-line filtration and etoposide phosphate. Given its water-soluble formulation and the absence of polysorbate 80 micelles, the mechanism of micelle destabilization described for conventional etoposide may be less relevant; however, this has not been formally investigated. On the other hand, patient-related characteristics such as age, sex and personal history of atopy do not appear to influence the risk of etoposide hypersensitivity in children substantially [5,20]. Management strategies for etoposide hypersensitivity in pediatric patients are largely based on the main risk factors identified in the literature—namely infusion-related variables, formulation characteristics, and prior exposure history. Acute reactions require immediate discontinuation of the infusion, clinical stabilization, and treatment with antihistamines, corticosteroids, and intramuscular epinephrine when clinically warranted [20,21]. When formulation-related mechanisms, particularly those linked to polysorbate 80, are suspected, switching from conventional etoposide to etoposide phosphate further decreases recurrence risk. Despite its improved excipient profile, etoposide phosphate is not routinely used as first-line therapy and is generally reserved for patients who develop hypersensitivity reactions to conventional etoposide. Its use may be influenced by practical considerations, including higher acquisition costs, limited availability, and occasional supply shortages, as well as more limited clinical experience and long-term pediatric outcome data, despite pharmacokinetic equivalence. Hypersensitivity reactions, although less frequent, have also been reported [38]. Premedication with antihistamines and corticosteroids is routinely used, especially in children with prior reactions, although its benefit seems greater for non-IgE-mediated mast-cell activation than for confirmed IgE-mediated hypersensitivity [5,17]. Finally, for patients who need ongoing therapy despite previous moderate or severe reactions, standardized rapid desensitization protocols have proven to be highly effective and safe across multiple pediatric case series. In this context, distinguishing between reaction types becomes clinically important: Type I hypersensitivity may require strict avoidance of the problematic formulation, comprehensive allergy testing if possible, and, when continuing therapy is necessary, the use of structured desensitization procedures. Type VII reactions, on the other hand, are more suitable for preventive strategies and can often be effectively managed by adjusting infusion conditions or switching to a polysorbate-free formulation. These customized approaches enable safe continuation of etoposide therapy while reducing the risk of recurrent reactions (Figure 2, Table 3).

## 5. Etoposide Desensitization Protocol

Desensitization to etoposide has become a strategy to maintain uninterrupted chemotherapy in pediatric patients who develop hypersensitivity reactions, which, although infrequent, can be severe or life-threatening. Drug desensitization is defined as a procedure that induces a temporary state of tolerance by administering progressively increasing doses of the culprit drug over a defined period of time, allowing safe re-exposure despite prior reactions. The literature describes various desensitization protocols, reflecting the diversity of clinical presentations and the need to adapt procedures to real-world constraints [19,39,40,41,42,43]. Despite this variability, the overarching principle remains consistent: gradual reintroduction of the drug through controlled, stepwise exposure at progressively increasing concentrations and rates, with the aim of attenuating mast cell and basophil reactivity and restoring tolerance. However, these protocols may be difficult to compare, as their structure is influenced by preparation methods, including dilution schemes and infusion systems. Therefore, the number of steps alone does not adequately characterize a desensitization protocol. A more appropriate comparison should be based on clinically relevant parameters, including stepwise dose escalation, number of administrations, duration of each infusion step, total infusion time, and cumulative dose delivered. Earlier protocols were generally characterized by prolonged infusion times and complex preparation procedures, particularly in patients with severe reactions (Table 4). For example, protocols described by Wright et al. involved 13–15 steps, multiple dilutions, and infusion times up to approximately 9.8 h, while Martinez et al. reported similarly structured protocols with extended duration and full premedication, reflecting a conservative and resource-intensive approach [39,40]. These approaches achieved high success rates but required extensive coordination between pharmacy, nursing, and medical teams, as well as significant resource utilization. More recent protocols have aimed to simplify preparation and reduce infusion duration while maintaining safety. Protocols described by Diebert et al. retained stepwise dose escalation but used fewer dilutions and shorter infusion times (approximately 4–6 h), improving feasibility without compromising effectiveness [41]. Similar time-efficient approaches have also been reported in smaller pediatric case series, including those by Turgay Yagmur et al., although these were not specifically designed as protocol-optimization studies [19]. These approaches rely on streamlined workflows and are more suitable for routine clinical practice, especially in pediatric and outpatient settings. In this context, differences between protocols are better understood in terms of infusion duration, number of administrations, and operational requirements rather than the number of steps alone. For instance, prolonged desensitization models such as the six-bag protocol reported by Stockton et al., with infusion times up to 29 h, illustrate the substantial impact on pharmacy workload, inpatient bed availability, and staffing despite high success rates [43]. Overall, despite structural variability, desensitization protocols share common mechanistic principles and demonstrate consistently high success rates when applied appropriately. Their clinical utility lies in enabling continuation of first-line therapy while balancing safety, feasibility, and resource utilization across different healthcare settings. These findings highlight that protocol complexity is driven not only by the number of steps but also by total infusion duration and operational requirements. Future efforts should focus on harmonizing desensitization strategies based on standardized clinical parameters rather than protocol structure alone, in order to improve comparability, reproducibility, and clinical applicability across centers.

## 6. Conclusions

In conclusion, current evidence shows that the rate of etoposide hypersensitivity in pediatric patients is much higher than previously thought, highlighting the need for increased clinical awareness. Reactions vary according to disease and treatment context, are usually immediate and mild to moderate in severity with predominantly cutaneous and respiratory features, but severe reactions, including anaphylaxis, may occur and require prompt management. Current evidence supports a heterogeneous pathophysiology. While confirmed IgE-mediated type I hypersensitivity appears to be rare in children, the majority of reactions are consistent with non-IgE-mediated mechanisms, likely related to formulation excipients such as polysorbate 80. Accurate differentiation between true hypersensitivity and infusion-related reactions, such as ethanol intoxication, is essential, as it directly influences preventive and therapeutic decisions. Preventive and therapeutic strategies for etoposide hypersensitivity should be tailored according to the presumed underlying mechanism of the reaction. Patients whose reactions are consistent with non-IgE-mediated mechanisms generally benefit from modification of administration-related factors, including optimization of infusion rates and, when feasible, switching to polysorbate-free formulations such as etoposide phosphate or oral etoposide. In these cases, avoidance of the offending excipient is often sufficient to prevent recurrence, and desensitization is usually not required. In contrast, patients with clinical features suggestive of true IgE-mediated hypersensitivity, supported by positive in vivo and in vitro testing, reproducibility of symptoms upon re-exposure, or severe immediate reactions, require a different management approach. For this subgroup, simple formulation changes may be inadequate, and rapid drug desensitization protocols represent the preferred strategy to allow safe continuation of therapy. Recognizing the underlying mechanism is therefore crucial to maintaining oncologic treatment intensity. At present, no pediatric studies have specifically evaluated the impact of etoposide discontinuation or substitution with alternative agents on survival outcomes. However, because etoposide represents a backbone component of several frontline pediatric chemotherapy protocols, preserving treatment continuity and avoiding unnecessary treatment interruptions remain important clinical objectives to maintain protocol-defined treatment intensity and planned cumulative drug exposure.

## Figures and Tables

**Table 2:** Definitions and Grading of Allergic and Infusion-Related Adverse Events According to CTCAE v6.0.

Adverse Event	Definition (CTCAE v6.0)	Grading (CTCAE v6.0)
Allergic Reaction/Hypersensitivity	A disorder characterized by an adverse general response from exposure to an allergen.	Grade 1: Systemic intervention not indicated Grade 2: Oral intervention indicated Grade 3: Bronchospasm; hospitalization; IV intervention Grade 4: Life-threatening; urgent intervention indicated Grade 5: Death
Anaphylaxis	A disorder characterized by an acute inflammatory reaction with breathing difficulty, hypotension, cyanosis, loss of consciousness.	Grade 1: – Grade 2: – Grade 3: Symptomatic bronchospasm; parenteral intervention; hypotension Grade 4: Life-threatening; urgent intervention Grade 5: Death
General Adverse Reactions	A disorder characterized by adverse reaction to infusion of pharmacological or biological substances.	Grade 1: Mild transient; no interruption Grade 2: Infusion interruption indicated; responds to treatment Grade 3: Prolonged/recurrent reaction; hospitalization Grade 4: Life-threatening; urgent intervention Grade 5: Death

**Table 3:** Practical management framework for acute reactions to intravenous etoposide in pediatric patients.

Phase	Governance Objective	Key Actions
Pre-infusion	Ensure safe administration	Verify formulation and infusion protocol; ensure monitoring and emergency medications
Acute reaction	Immediate patient safety	Interrupt infusion; assess severity; provide supportive treatment
Diagnostic evaluation	Identify reaction mechanism	Differentiate IgE-mediated and non- IgE-mediated hypersensitivity from infusion-related adverse events
Treatment strategy	Preserve protocol-defined therapy	Consider infusion optimization, alternative formulation (etoposide phosphate), or desensitization

**Table 4:** Summary of Etoposide Desensitization Protocols in Pediatric Patients.

	Patients	Premedication	Steps	Concentration Bags System	Duration	Success Rate
Stockton et al. (2021) [43]	12 patients	Steroids, H1/H2 blockers, albuterol	6 consecutive bags	N.A.	9–15 h Up to 29 h in selected patients	11/12 (92%)
Diebert et al. (2020) [41]	N.A.	H1/H2 blockers, steroids, montelukast (night before)	3 syringes/4 rate changes each (12 total rate-steps)	Syringe 1 (1:100): 0.003 mg/mL Syringe 2 (1:10): 0.03 mg/mL Bag 3 (1:1): 0.3 mg/mL (Four rate increases every 15 min)	6 h	3 doses over 3 consecutive days for a total 9 doses
Turgay Yagmur et al. (2020) [19]	3 patients	Hydroxyzine, methylprednisolone	12 steps and 3 bag method	Bag 1: 0.002 mg/mL Bag 2: 0.02 mg/mL Bag 3: 0.2 mg/m	4 h	100%
Martinez et al. (2020) [40]	1 patient	Night before: montelukast, prednisone Before infusion: montelukast, diphenhydramine IV, albuterol, methylprednisolone IV(converted from prednisone)	12-step protocol (1 h per bag, rate doubled every 15 min)	Bag 1: 0.003 mg/mL Bag 2: 0.03 mg/mL Bag 3: 0.3 mg/mL	6 h	100%
Tara E. Wright et al. (2019) [39]	9 patients	Cetirizine, ranitidine PO (24 h before), diphenhydramine IV (30 min before), ranitidine PO (30 min before), hydrocortisone IV (30 min before)	13-step protocol (most patients) 15-step protocol (used in 1 patient after reaction to 13-step)	13-step protocol Bag 1: 0.00276 mg/mL Bag 2: 0.0276 mg/mL Bag 3: 0.276 mg/mL 15-step protocol Bag 1: 0.0034 mg/mL Bag 2: 0.034 mg/mL Bag 3: 0.34 mg/mL	13 steps 7 h 15 steps 9.8 h	13-step: 8/9 patients tolerated (89%) 15-step: 1/1 successful
Kulhas Celik I. et al. (2018) [42]	1 child aged 2.5 years	Hydroxyzine PO, methylprednisolone IV	12 steps and 3 solutions: first solution: 0.002 mg/mL, second solution: 0.02 mg/mL, third solution: 0.2 mg/mL		4 h	100%

IV: intravenous. PO: oral route. N.A.: not available.

## Data Availability

No new data were created or analyzed in this study. Data sharing is not applicable to this article.

## Abbreviations

The following abbreviations are used in this manuscript:

ALDH	Aldehyde dehydrogenase
ALL	Acute lymphoblastic leukemia
AIFA	Italian Medicines Agency
BAT	Basophil activation test
CTCAE	Common Terminology Criteria for Adverse Events
EAACI	European Academy of Allergy and Clinical Immunology
EMA	European Medicines Agency
FcεRI	High-affinity immunoglobulin E receptor
HSCT	Hematopoietic stem cell transplantation
HSR	Hypersensitivity reaction
ICU	Intensive care unit
IgE	Immunoglobulin E
IgG	Immunoglobulin G
ILF	In-line filter
IV	Intravenous
N.A.	Not available
PEG	Polyethylene glycol
PS80	Polysorbate 80
VP16	Etoposide
